# Biomimetic Linkage Mechanism Robust Control for Variable Stator Vanes in Aero-Engine

**DOI:** 10.3390/biomimetics9120778

**Published:** 2024-12-21

**Authors:** Qinqin Sun, Zhangyang Lu, Xingyu Gui, Ye-Hwa Chen

**Affiliations:** 1 School of Energy and Power Engineering, Nanjing University of Aeronautics and Astronautics, Nanjing 210016, China; qinqinsun@nuaa.edu.cn (Q.S.); luzhangyangsz2202061@nuaa.edu.cn (Z.L.); guixingyu@nuaa.edu.cn (X.G.); 2 The George W. Woodruff School of Mechanical Engineering, Georgia Institute of Technology, Atlanta, GA 30332, USA

**Keywords:** matching condition, uncertain nonlinear system, time-varying uncertainty, robust control, electro-hydrostatic actuator

## Abstract

This work addresses the position tracking control design of the stator vane driven by electro-hydrostatic actuators facing uncertain aerodynamic disturbances. Rapidly changing aerodynamic conditions impose complex disturbance torques on the guide vanes. Consequently, a challenging task is to enhance control precision in complex uncertain environments. Inspired by the principles of mammalian muscle movement, a novel robust control strategy based on the backstepping method has been proposed. Using backstepping, virtual rotational speed and virtual pressure difference force are designed, which decompose the high-order position closed-loop control problem into three lower-order parts, eliminating the need for matching conditions. Subsequently, robust controllers were designed, and stability proofs and performance analyses of the controllers were provided. This control strategy was tested through numerical hydraulic simulation. The results show that compared to other control methods, this approach significantly improves tracking accuracy and robustness. Therefore, it is believed that this method has the potential to become a new generation solution for such problems.

## 1. Introduction

With the development of materials and sensors science [1,2], aircraft engines are gradually moving towards a multivariable and distributed control architecture [3], local closed-loop control of actuators and sensors in aero-engines are gradually gaining attention [4,5,6]. Stator vane control is used to address the surge issue in aero-engines. The variable inlet guide vanes at the low-pressure compressor inlet are connected to electro-hydraulic actuators via a linkage mechanism. Therefore, by changing the input electrical signals to the electro-hydraulic actuators, the angle of attack of the stator vanes can be altered, thereby improving the airflow state entering the high-pressure compressor.

The challenge of stator vane control lies in balancing power output with precision. The airflow at the compressor inlet is easily affected by flight conditions, making the stator vanes subject to uncertain aerodynamic torque disturbances. Simply increasing the output power of the electro-hydraulic actuators can reduce the impact of uncertainty to some extent, but an excessive increase in control cost brings the risks of stator vane flutter and overheating of the electro-hydraulic actuators. On the other hand, insufficient output power may not generate enough pressure difference in the hydraulic cylinder to resist external load forces, making the stator vanes more susceptible to aerodynamic torque disturbances.

**Figure 1:**
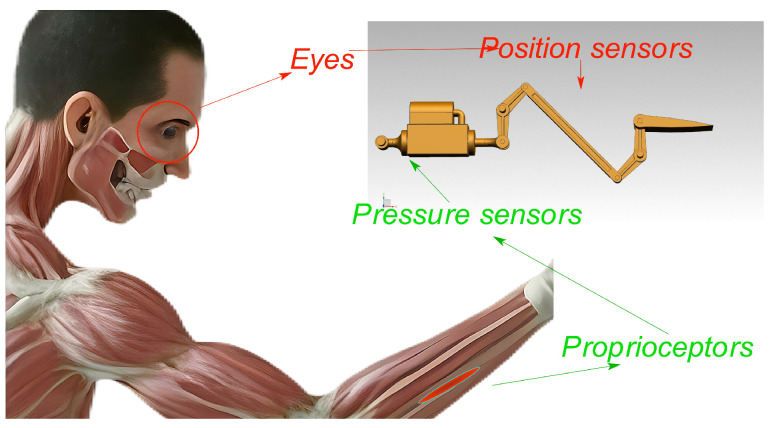
Insights from the Biological Nervous Control System.

To address such issues, perhaps we can draw experience from biology. Muscle spindles and Golgi tendon organs, as proprioceptors, are widely present in the muscle and connective tissues of mammals and are highly sensitive to changes in muscle strength and length [7]. When mammals perform voluntary movements such as grasping, proprioceptors and exteroceptors work together. Proprioceptors send information about muscle movement into the central nervous system, regulating the rate and acceleration of movement to maintain stability, while visual and other exteroceptor signals are also input into the central nervous system to further refine spatial position and other movement details. It is widely believed that these two processes cannot be completed independently [8,9]. Voluntary movement in mammals is a multi-closed-loop control process, and the complex closed-loop synergy between proprioceptors and exteroceptors is key to achieving fine movement control in mammals. As is shown in Figure 1, based on a similar principle of action, introducing additional closed loops beyond the position closed loop into the stator vane control system may improve the precision of stator vane control. To address this issue, a more flexible control algorithm is required that can maintain control performance as much as possible under aerodynamic torque disturbances.

However, implementing pressure feedback at the control law level brings new problems. In most cases, whether it is a pressure closed loop or a position closed loop, it can only be achieved by adjusting the hydraulic cylinder’s import flow, and the input signal requirements for the two closed-loop controls can easily conflict. Therefore, the synergy of multi-closed-loop control must be considered. A common method is to use the backstepping method to construct virtual control. First, the expected pressure difference required for position control is calculated in real-time based on the actual position of the hydraulic cylinder, and then the pressure closed-loop control is used to reduce the difference between the expected and actual pressure differences. From a control theory perspective, such a multi-closed-loop control structure can transform the originally non-matching uncertainty [10] into matching by adding virtual control to the differential equations not directly controlled, to suppress the impact of uncertainty.In recent years, many controllers using the backstepping strategy have been proposed [11,12,13,14].

However, for the stator vane control problem, there is still a final challenge: how to deal with uncertainties that may change rapidly over time. For example, due to the complex flow field near the blades inside the aero-engine, it is difficult to assume that the aerodynamic torque uncertainty of the blades is slowly varying. This poses higher requirements for controller performance.

Designing a controller that can make such a system asymptotically stable is a very challenging task. However, in practical applications, people do not always need to insist on achieving a complete zero error. By tolerating a sufficiently small error, the concept of “practical stability” has been proposed [15] and a series of related studies have been conducted [16,17,18,19,20,21]. To ensure that state variables can converge to a sufficiently small value within a finite time in the presence of rapidly varying disturbances [22,23,24,25], we propose a robust control based on the backstepping method within the framework of practical stability. The control has three control loops: pressure, speed, and position, to ensure that all uncertainties can be directly affected by control. We also made a quantitative analysis of the control performance and conducted simulation verification.

This paper has three main contributions. First, a general mathematical model of the aero-engine stator vane angle control system is established. The model takes into account various mismatches and stricter uncertainties in external forces and internal actuators. Second, a robust controller based on the mathematical model is designed. The controller can effectively handle various complex uncertainties. In theory, it ensures that errors caused by disturbances can converge to a sufficiently small level within a finite time. Third, the control performance in various environments has been verified through simulation, proving that this method has the potential to become a new solution for such servo control problems.

## 2. Model

**Figure 2:**
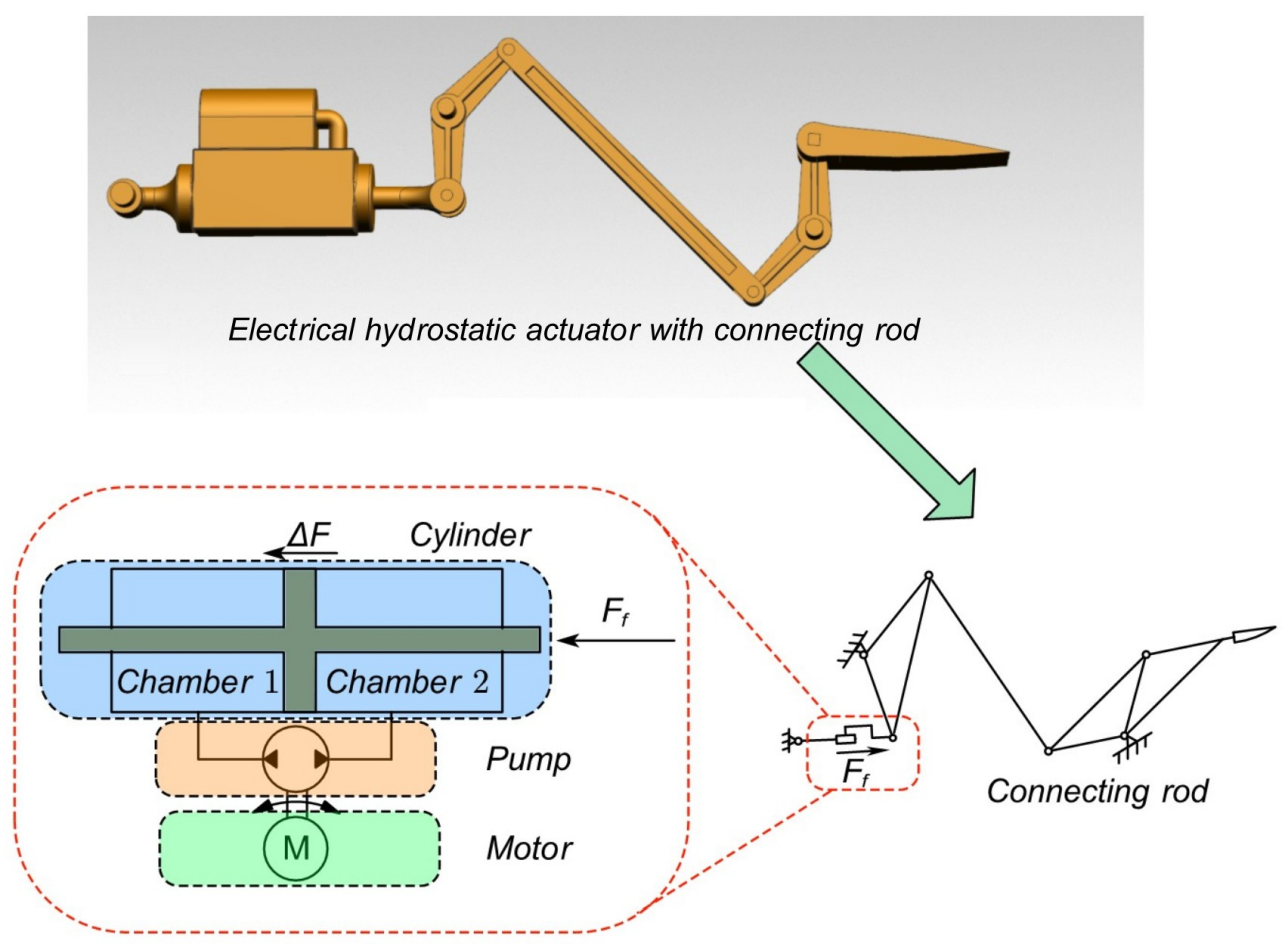
The Structure of the Guide Vane Control Mechanism.

An aero-engine electro-hydrostatic actuator consists of an electric pump and a cylinder subjected to external forces from various transmission mechanisms. As shown in Figure 2, the hydraulic oil from the pump enters the cylinder to provide power to the servo system, actuating the piston and the rod.

Consider the dynamic equations of the electro-hydrostatic actuator. The pressure difference force on the hydraulic piston, the external load force on the push rod, and the friction force inside the hydraulic cylinder collectively affect the motion of the piston, thereby obtaining:

(1) $$\begin{array}{c} m\ddot{y}\left(t\right)=S_{1}P_{1}\left(t\right)-S_{2}P_{2}\left(t\right)-B_{p}\dot{y}\left(t\right)-F_{f}\left(y\left(t\right),\dot{y}\left(t\right),t\right)-\Delta F\left(t\right), \end{array}$$

where *t* is the time, m>0 is the total mass of the pushrod-piston system, $S_{1}>0$ and $S_{2}>0$ are the areas of the two ends of the piston, $B_{p}>0$ is the viscous friction coefficient, $P_{1}\left(t\right)$ and $P_{2}\left(t\right)$ are the pressure of chamber 1 and 2, y(t) is the length of chamber 1, $\dot{y}\left(t\right)$ and $\ddot{y}\left(t\right)$ are the first- and second-order derivatives of y(t) with respect to time, $F_{f}\left(y\left(t\right),\dot{y}\left(t\right),t\right)$ is the external loading force at the end of the pushrod and ΔF(t) is the friction.

From [26], the pressure exerted on the piston is determined by the load flow rate of the hydraulic cylinder, which results in:

(2) $$\begin{array}{c} \begin{array}{c} {\dot{P}}_{1}\left(t\right)=\frac{\beta_{e}}{V_{0}+S_{1}\left|y(t)\right|}\left(-S_{1}\dot{y}\left(t\right)-\Delta Q_{c1}\left(t\right)+Q_{L}\left(t\right)\right)\\ {\dot{P}}_{2}\left(t\right)=\frac{\beta_{e}}{V_{0}+S_{2}\left|\left(l-y(t)\right)\right|}\left(S_{2}\dot{y}\left(t\right)+\Delta Q_{c2}\left(t\right)-Q_{L}\left(t\right)\right), \end{array} \end{array}$$

where $\beta_{e}$ is the bulk modulus of liquid, $V_{0}>0$ is the dead volume, $\Delta Q_{c1}\left(t\right)$ and $\Delta Q_{c2}\left(t\right)$ are external leakage of two chambers, *l* is the length of the cylinder and $Q_{L}\left(x_{v},P_{1},t\right)$ is the load flow rate. Therefore,

(3) $$\begin{array}{cc} \frac{d(S_{1}P_{1}\left(t\right)-S_{2}P_{2}\left(t\right))}{dt}= & -\left(\frac{\beta_{e}}{\frac{V_{0}}{S_{1}}+\left|y(t)\right|}S_{1}+\frac{\beta_{e}}{\frac{V_{0}}{S_{2}}+\left|l-y(t)\right|}S_{2}\right)\dot{y}\left(t\right)\\ \; & -\frac{\beta_{e}}{\frac{V_{0}}{S_{1}}+\left|y(t)\right|}\Delta Q_{c1}\left(t\right)-\frac{\beta_{e}}{\frac{V_{0}}{S_{2}}+\left|l-y(t)\right|}\Delta Q_{c2}\left(t\right)\\ \; & +\left(\frac{\beta_{e}}{\frac{V_{0}}{S_{1}}+\left|y(t)\right|}+\frac{\beta_{e}}{\frac{V_{0}}{S_{2}}+\left|l-y(t)\right|}\right)Q_{L}\left(t\right). \end{array}$$

The load flow rate of the hydraulic cylinder can be determined by changing the rotation speed and displacement of the electric pump. For instance, with a constant displacement pump, the load flow rate of the hydraulic cylinder is directly influenced by the rotation speed of the constant displacement pump. Therefore,

(4) $$\begin{array}{c} Q_{L}\left(t\right)=\Delta d_{q}\left(t\right)+k_{q}\omega \left(t\right), \end{array}$$

where $\Delta d_{q}\left(t\right)$ is the flow rate error caused by pump leakage, $k_{q}$ is the pump displacement and ω(t) is the rotation speed of the electric pump. The rotation speed is determined by the input voltage of the motor. Therefore,

(5) $$\begin{array}{c} \dot{\omega }\left(t\right)=\frac{k_{m}\left(t\right)}{J_{a}}u\left(t\right)-\frac{\Delta T_{b}\left(t\right)}{J_{a}}-\frac{B_{m}\omega \left(t\right)}{J_{a}}, \end{array}$$

where $k_{m}\left(t\right)$ is the motor torque coefficient, $\Delta T_{b}\left(t\right)$ is the combined load torque, $B_{m}$ is the combined viscous damping and $J_{a}$ is the rotational inertia. For the ease of control law design, rewrite the dynamic equation of the electro-hydrostatic actuator in another form. Selecting the state variables

(6) $$\begin{array}{c} \begin{array}{c} x_{11}\left(t\right)=my\left(t\right)-my_{d}\left(t\right),\\ x_{12}\left(t\right)=m\dot{y}\left(t\right)-m{\dot{y}}_{d}\left(t\right),\\ x_{2}\left(t\right)=P_{1}\left(t\right)S_{1}-P_{2}\left(t\right)S_{2},\\ x_{3}\left(t\right)=\omega \left(t\right), \end{array} \end{array}$$

where $y_{d}\left(t\right)$ is the instruction signal that is three times continuously differentiable with respect to *t*, to obtain the state-space equation

(7) $$\begin{array}{cc} {\dot{x}}_{11}\left(t\right)= & x_{12}\left(t\right),\\ {\dot{x}}_{12}\left(t\right)= & -m{\ddot{y}}_{d}\left(t\right)-B_{p}\dot{y}\left(t\right)-F_{f}\left(y\left(t\right),\dot{y}\left(t\right),t\right)-\Delta F\left(t\right)+x_{2}\left(t\right)\\ {\dot{x}}_{2}\left(t\right)= & -\left[L_{1}\left(y\left(t\right)\right)S_{1}+L_{2}\left(y\left(t\right)\right)S_{2}\right]\dot{y}\left(t\right)-L_{1}\left(y\left(t\right)\right)\Delta Q_{c1}\left(t\right)-L_{2}\left(y\left(t\right)\right)\Delta Q_{c2}\left(t\right)\\ \; & +\left[L_{1}\left(y\left(t\right)\right)+L_{2}\left(y\left(t\right)\right)\right]\Delta d_{q}\left(t\right)+\left[L_{1}\left(y\left(t\right)\right)+L_{2}\left(y\left(t\right)\right)\right]k_{q}x_{3}\left(t\right)\\ {\dot{x}}_{3}\left(t\right)= & -\frac{x_{3}\left(t\right)}{J_{a}}-\frac{\Delta T_{b}\left(t\right)}{J_{a}}+\frac{k_{m}\left(t\right)}{J_{a}}u\left(t\right), \end{array}$$

where

(8) $$\begin{array}{cc} L_{1}\left(y\left(t\right)\right) & =\frac{\beta_{e}}{\frac{V_{0}}{S_{1}}+\left|y(t)\right|}, \end{array}$$

(9) $$\begin{array}{cc} L_{2}\left(y\left(t\right)\right) & =\frac{\beta_{e}}{\frac{V_{0}}{S_{2}}+\left|l-y(t)\right|}. \end{array}$$

Considering friction and uncertain load forces, therefore choose $F_{f}\left(y\left(t\right),\dot{y}\left(t\right),t\right)={\bar{F}}_{f}\left(y\left(t\right),\dot{y}\left(t\right),t\right)+\Delta F_{f}\left(t\right)$. In this context, ${\bar{F}}_{f}\left(y\left(t\right),\dot{y}\left(t\right),t\right)$ represents the external load force derived from theoretical calculations, while $\Delta F_{f}\left(t\right)$ represents the uncertain but bounded external load disturbance. Therefore, from (7),

(10) $$\begin{array}{cc} {\dot{x}}_{11}\left(t\right)= & x_{12}\left(t\right)\\ {\dot{x}}_{12}\left(t\right)= & -m{\ddot{y}}_{d}\left(t\right)-B_{p}\dot{y}\left(t\right)-{\bar{F}}_{f}\left(y\left(t\right),\dot{y}\left(t\right),t\right)-\Delta F_{f}\left(t\right)-\Delta F\left(t\right)+x_{2}\left(t\right)\\ = & -k_{11}x_{11}\left(t\right)-k_{12}x_{12}\left(t\right)+k_{11}x_{11}\left(t\right)+k_{12}x_{12}\left(t\right)-m{\ddot{y}}_{d}\left(t\right)-B_{p}\dot{y}\left(t\right)\\ \; & -{\bar{F}}_{f}\left(y\left(t\right),\dot{y}\left(t\right),t\right)-\Delta F_{f}\left(t\right)-\Delta F\left(t\right)+x_{2}\left(t\right), \end{array}$$

in which $k_{11}>0$ and $k_{12}>0$ are parameters that can be selected. For details on how to choose these parameters to improve system performance, see Section 4. Thus from (10), obtained subsystem

(11) $$\begin{array}{c} \left(S_{1}\right):{\dot{x}}_{1}\left(t\right)=f_{1}\left(x_{1}\left(t\right)\right)+\Delta f_{1}\left(x_{1}\left(t\right),\sigma_{1}\left(t\right),t\right)+\left(B_{1}+\Delta B_{1}\right)x_{2}\left(t\right), \end{array}$$

(12) $$\begin{array}{c} x_{1}\left(t\right)={\left[x_{11}\left(t\right),x_{12}\left(t\right)\right]}^{T}, \end{array}$$

(13) $$\begin{array}{c} \sigma_{1}\left(t\right)={\left[\Delta F_{f}\left(t\right),\Delta F\left(t\right)\right]}^{T}, \end{array}$$

(14) $$\begin{array}{c} f_{1}\left(x_{1}\left(t\right)\right)=\left[\begin{array}{c} x_{12}\left(t\right)\\ -k_{11}x_{11}\left(t\right)-k_{12}x_{12}\left(t\right) \end{array}\right], \end{array}$$

(15) $$\begin{array}{c} \Delta f_{1}\left(x_{1}\left(t\right),\sigma_{1}\left(t\right),t\right)=\left[\begin{array}{c} 0\\ \Delta f_{12}\left(x_{1}\left(t\right),\sigma_{1}\left(t\right),t\right) \end{array}\right],\\ \Delta f_{12}\left(x_{1}\left(t\right),\sigma_{1}\left(t\right),t\right)=k_{11}x_{11}\left(t\right)+k_{12}x_{12}\left(t\right)-m{\ddot{y}}_{d}\left(t\right)-B_{p}\dot{y}\left(t\right) \end{array}$$

(16) $$\begin{array}{c} -{\bar{F}}_{f}\left(y\left(t\right),\dot{y}\left(t\right),t\right)-\Delta F_{f}\left(t\right)-\Delta F\left(t\right), \end{array}$$

(17) $$\begin{array}{c} B_{1}=1, \end{array}$$

(18) $$\begin{array}{c} \Delta B_{1}=0. \end{array}$$

**Remark** **1.** *In fact, the $f_{i}\left(\cdot \right)$ here is not part of the original model, but is artificially selected to enhance control performance. The quantitative relationship between $f_{i}\left(\cdot \right)$ and control performance will be discussed in Section 4.*

Considering that unpredictable leaks will affect the pressure changes in both chambers of the hydraulic cylinder, uncertain leakage flow rate $\Delta Q_{c1}\left(t\right),\Delta Q_{c2}\left(t\right)$, pump flow rate error $\Delta d_{q}\left(t\right)$ and the flow error caused by temperature changes ΔQ(t) are therefore considered. Thus from (7), obtained subsystem

(19) $$\begin{array}{cc} \left(S_{2}\right):{\dot{x}}_{2}\left(t\right)= & f_{2}\left(x_{2}\left(t\right)\right)+\Delta f_{2}\left(x_{2}\left(t\right),\sigma_{2}\left(t\right),t\right)+\left[B_{2}\left(t\right)+\Delta B_{2}\right]x_{3}\left(t\right), \end{array}$$

(20) $$\begin{array}{cc} \sigma_{2}\left(t\right)= & {\left[\Delta Q_{c1}\left(t\right),\Delta Q_{c2}\left(t\right),\Delta d_{q}\left(t\right),\Delta Q\left(t\right)\right]}^{T}, \end{array}$$

(21) $$\begin{array}{cc} f_{2}\left(x_{2}\left(t\right)\right)= & -k_{2}x_{2}\left(t\right), \end{array}$$

(22) $$\begin{array}{cc} \Delta f_{2}\left(x_{2}\left(t\right),\sigma_{2}\left(t\right),t\right)= & k_{2}x_{2}\left(t\right)-\left[L_{1}\left(y\left(t\right)\right)S_{1}+L_{2}\left(y\left(t\right)\right)S_{2}\right]\dot{y}\left(t\right)-L_{1}\left(y\left(t\right)\right)\Delta Q_{c1}\left(t\right)\\ \; & -L_{2}\left(y\left(t\right)\right)\Delta Q_{c2}\left(t\right)+\left[L_{1}\left(y\left(t\right)\right)+L_{2}\left(y\left(t\right)\right)\right]\Delta d_{q}\left(t\right)+\Delta Q\left(t\right), \end{array}$$

(23) $$\begin{array}{cc} B_{2}\left(t\right)= & \left[L_{1}\left(y\left(t\right)\right)+L_{2}\left(y\left(t\right)\right)\right]k_{q}, \end{array}$$

(24) $$\begin{array}{cc} \Delta B_{2}= & 0, \end{array}$$

in which $k_{2}>0$ is a parameter that can be selected.

Considering the unpredictable loading torque $\Delta T_{b}\left(t\right)$ and motor torque coefficient $k_{m}\left(t\right)={\bar{k}}_{m}+\Delta k_{m}\left(t\right)$, thus from (7) obtained subsystem

(25) $$\begin{array}{cc} \left(S_{3}\right):{\dot{x}}_{3}\left(t\right)= & f_{3}\left(x_{3}\left(t\right)\right)+\Delta f_{3}\left(x_{3}\left(t\right),\sigma_{3}\left(t\right),t\right)+\left[B_{3}+\Delta B_{3}\left(t\right)\right]u\left(t\right), \end{array}$$

(26) $$\begin{array}{cc} \sigma_{3}\left(t\right)= & {\left[\Delta T_{b}\left(t\right),\Delta k_{m}\left(t\right)\right]}^{T}, \end{array}$$

(27) $$\begin{array}{cc} f_{3}\left(x_{3}\left(t\right)\right)= & -k_{3}x_{3}\left(t\right), \end{array}$$

(28) $$\begin{array}{cc} \Delta f_{3}\left(x_{3}\left(t\right),\sigma_{3}\left(t\right),t\right)= & k_{3}x_{3}\left(t\right)-\frac{x_{3}\left(t\right)}{J_{a}}-\frac{\Delta T_{b}\left(t\right)}{J_{a}}, \end{array}$$

(29) $$\begin{array}{cc} B_{3}= & \frac{{\bar{k}}_{m}}{J_{a}}, \end{array}$$

(30) $$\begin{array}{cc} \Delta B_{3}\left(t\right)= & \frac{\Delta k_{m}\left(t\right)}{J_{a}}, \end{array}$$

in which $k_{3}>0$ is a parameter that can be selected.

## 3. Controller

Considering the following system:

(31) $$\begin{array}{cc} \left(S_{1}\right){\dot{x}}_{1}\left(t\right)= & f_{1}\left(x_{1}\left(t\right),t\right)+\Delta f_{1}\left(x\left(t\right),\sigma_{1}\left(t\right),t\right)\\ \; & +\left[B_{1}\left(x_{1}\left(t\right),t\right)+\Delta B_{1}\left(x\left(t\right),\sigma_{1}\left(t\right),t\right)\right]x_{2}\left(t\right),\\ \left(S_{2}\right){\dot{x}}_{2}\left(t\right)= & f_{2}\left(x_{2}\left(t\right),t\right)+\Delta f_{2}\left(x\left(t\right),\sigma_{2}\left(t\right),t\right)\\ \; & +\left[B_{2}\left(x_{2}\left(t\right),t\right)+\Delta B_{2}\left(x\left(t\right),\sigma_{2}\left(t\right),t\right)\right]x_{3}\left(t\right),\\ \vdots \\ \left(S_{i}\right){\dot{x}}_{i}\left(t\right)= & f_{i}\left(x_{i}\left(t\right),t\right)+\Delta f_{i}\left(x\left(t\right),\sigma_{i}\left(t\right),t\right)\\ \; & +\left[B_{i}\left(x\left(t\right),t\right)+\Delta B_{i}\left(x\left(t\right),\sigma_{i}\left(t\right),t\right)\right]x_{i+1}\left(t\right),\\ \vdots \\ \left(S_{N}\right){\dot{x}}_{N}\left(t\right)= & f_{N}\left(x_{N}\left(t\right),t\right)+\Delta f_{N}\left(x\left(t\right),\sigma_{N}\left(t\right),t\right)\\ \; & +\left[B_{N}\left(x\left(t\right),t\right)+\Delta B_{N}\left(x\left(t\right),\sigma_{N}\left(t\right),t\right)\right]u\left(t\right), \end{array}$$

where t∈R is the time, $N\in N_{+}$ and N>1, i=1,2,…,N−1, $x_{i}\left(t\right)\in R^{n_{i}}$ is the state, $x\left(t\right)={\left[x_{1}^{T}\left(t\right),x_{2}^{T}\left(t\right),\ldots ,x_{N}^{T}\left(t\right)\right]}^{T}\in R^{n},n=\sum_{i=1}^{N}n_{i}$, $\sigma_{i}\in \Sigma_{i}\subset R^{s_{i}}$ is the uncertainty, $u\left(t\right)\in R^{m}$ is the input of the system. The system vectors and matrices $\Delta f_{i}\left(\cdot \right):R^{n}\times R^{s_{i}}\times R\to R^{n_{i}}$, i=1,2,…,N, $B_{i}\left(\cdot \right):R^{n_{i}}\times R\to R^{n_{i}}\times R^{n_{i+1}}$, $\Delta B_{i}\left(\cdot \right):R^{n}\times R^{s_{i}}\times R\to R^{n_{i}}\times R^{n_{i+1}}$, i=1,2,…,N−1, $B_{N}\left(\cdot \right):R^{n_{N}}\times R\to R^{n_{N}}\times R^{m}$ and $\Delta B_{N}\left(\cdot \right):R^{n}\times R^{s_{N}}\times R\to R^{n_{N}}\times R^{m}$ are continuous.

The control object is to design a u(t) to make state $x_{1}\left(t\right)$ practically stable. Strictly speaking, the system *S* under the control u(t) should meet the following practically stable conditions:

(i) Existence and Continuation of the solution: The system *S* possesses a solution $x_{1}\left(t\right):\left[t_{0},\infty \right)\to R^{n_{1}}$.
(ii) Uniform boundedness: For any $r_{z}>0$, there exist a constant $d_{z}\left(r_{z}\right)>0$ such that if $∥x_{1}\left(t_{0}\right)∥\leq r_{z}$, then $∥x_{1}\left(t\right)∥\leq d_{z}\left(r_{z}\right)$ for all $t\geq t_{0}$.
(iii) Uniform ultimate boundedness: There exists d>0, such that for any $\bar{d}>d$, any $r_{z}>0$ and any $x_{1}\left(t\right)$ with $∥x_{1}\left(t_{0}\right)∥\leq r_{z}$, there exist a finite time $T(\bar{d},r_{z})$ such that $∥x_{1}\left(t\right)∥\leq \bar{d}$ for any $t\geq t_{0}+T\left(\bar{d},r_{z}\right)$.

In order to obtain robust control based on the backstepping method, consider making the following state transformation first:

(32) $$\begin{array}{cc} z_{1}\left(t\right) & =x_{1}\left(t\right),\\ z_{i}\left(t\right) & =x_{i}\left(t\right)-u_{i-1}\left(t\right), \end{array}$$

i=2,3,…,N, where $u_{i-1}\left(t\right)$ is the virtual control and will be given later. This way, the state tracking control problem of a high-order system can be transformed into multiple state tracking control problems of low-order systems. This is also the core idea of the backstepping method. From (31) and (32), the system after state transformation can be written as:

(33) $$\begin{array}{cc} \left({\hat{S}}_{1}\right){\dot{z}}_{1}\left(t\right)= & f_{1}\left(z_{1}\left(t\right)\right)+f_{1}\left(x_{1}\left(t\right)\right)-f_{1}\left(z_{1}\left(t\right)\right)+\Delta f_{1}\left(x\left(t\right),\sigma_{1}\left(t\right),t\right)\\ \; & +\left[B_{1}\left(x_{1}\left(t\right),t\right)+\Delta B_{1}\left(x\left(t\right),\sigma_{1}\left(t\right),t\right)\right]\left(z_{2}\left(t\right)+u_{1}\left(t\right)\right),\\ \left({\hat{S}}_{2}\right){\dot{z}}_{2}\left(t\right)= & f_{2}\left(z_{2}\left(t\right)\right)+f_{2}\left(x_{2}\left(t\right)\right)-f_{2}\left(z_{2}\left(t\right)\right)+\Delta f_{2}\left(x\left(t\right),\sigma \left(t\right),t\right)-{\dot{u}}_{1}\\ \; & +\left[B_{2}\left(x_{2}\left(t\right),t\right)+\Delta B_{2}\left(x\left(t\right),\sigma \left(t\right),t\right)\right]\left(z_{3}\left(t\right)+u_{2}\right),\\ \vdots \\ \left({\hat{S}}_{i}\right){\dot{z}}_{i}\left(t\right)= & f_{i}\left(z_{i}\left(t\right)\right)+f_{i}\left(x_{i}\left(t\right)\right)-f_{i}\left(z_{i}\left(t\right)\right)+\Delta f_{i}\left(x\left(t\right),\sigma_{i}\left(t\right),t\right)-{\dot{u}}_{i-1}\left(t\right)\\ \; & +\left[B_{i}\left(x_{i}\left(t\right),t\right)+\Delta B_{i}\left(x\left(t\right),\sigma_{i}\left(t\right),t\right)\right]\left(z_{i+1}\left(t\right)+u_{i}\right),\\ \vdots \\ \left({\hat{S}}_{N}\right){\dot{z}}_{N}\left(t\right)= & f_{N}\left(z_{N}\left(t\right)\right)+f_{N}\left(x_{N}\left(t\right)\right)-f_{N}\left(z_{N}\left(t\right)\right)+\Delta f_{N}\left(x\left(t\right),\sigma_{N}\left(t\right),t\right)-{\dot{u}}_{N-1}\left(t\right)\\ \; & +\left[B_{N}\left(x_{N}\left(t\right),t\right)+\Delta B_{N}\left(x\left(t\right),\sigma_{N}\left(t\right),t\right)\right]u, \end{array}$$

where $f_{i}\left(\cdot \right):R^{n_{i}}\times R\to R^{n_{i}}$ is a function that has been artificially designed based on Assumption 1.

**Assumption** **A1.** *$f_{i}\left(\cdot \right):R^{n_{i}}\to R^{n_{i}}$, i=1,2,…,N are the function satisfies that for systems ${\dot{x}}_{i}\left(t\right)=f_{i}\left(x_{i}\left(t\right)\right)$ at the origins $x_{i}=0$ mentioned at (31). Thus following conditions can be satisfied:*

*(1)*  *$f_{i}\left(0\right)=0$. and Continuation of the solution: The system S possesses a solution $x_{1}\left(t\right):\left[t_{0},\infty \right)\to R^{n_{1}}$.*
*(2)*  *There are continuous mappings $V_{i}\left(\cdot \right):R^{n_{i}}\to R_{+}$, i=1,2,…,N, the continuous, strictly increasing functions $\gamma_{ji}\left(\cdot \right):R_{+}\to R_{+}$, j=1,2 which satisfy $\gamma_{ji}\left(0\right)=0$, $\lim_{r\to \infty }\gamma_{ji}\left(r\right)=\infty$ for all r∈R, and a positive constant $\gamma_{3i}$, such that for all $x_{i}\in R^{n_{i}}$,*
(34) $$\begin{array}{c} \gamma_{1i}\left(∥x_{i}∥\right)\leq V_{i}\left(x_{i}\right)\leq \gamma_{2i}\left(∥x_{i}∥\right), \end{array}$$
(35) $$\begin{array}{cc} L_{0}^{i}\left(x_{i}\right):= & \frac{\partial V_{i}\left(x_{i}\right)}{\partial t}+\nabla_{x_{i}}^{T}V_{i}\left(x_{i}\right)f_{i}\left(x_{i}\right)\leq -\gamma_{3i}{∥x_{i}∥}^{2}. \end{array}$$

Additionally, it is assumed that the transformed system should also satisfies the following hypothetical conditions:

**Assumption** **2.** *There exist mappings $h_{i}\left(\cdot \right):R^{n}\times R^{n}\times R^{s_{i}}\times R\to R^{n_{i+1}}$, i=1,2,…,N−1, mapping $h_{i}\left(\cdot \right):R^{n}\times R^{n}\times R^{s_{N}}\times R\to R^{m}$, mappings $E_{i}\left(\cdot \right):R^{n}\times R^{s_{i}}\times R\to R^{n_{i}\times n_{i+1}}$, i=1,2,…,N−1 and mapping $E_{N}\left(\cdot \right):R^{n}\times R^{s_{i}}\times R\to R^{n_{N}\times m}$ such that for all $x\in R^{n}$, $\sigma_{i}\in \Sigma_{i}$, $u\in R^{m}$ and t∈R,*

(36) $$\begin{array}{cc} f_{i}\left(x_{i}\right)-f_{i}\left(z_{i}\right)+\Delta f_{i}\left(x,\sigma_{i},t\right)-{\dot{u}}_{i-1}\left(t\right) & =B_{i}\left(x_{i}\right)h_{i}\left(z,x,\sigma_{i},t\right), \end{array}$$

(37) $$\begin{array}{cc} \Delta B_{i}\left(x,\sigma_{i},t\right) & =B_{i}\left(x_{i}\right)E_{i}\left(z,x,\sigma_{i},t\right), \end{array}$$

*i=1,2,…,N. Furthermore, there exist continuous mapping $\rho_{h_{i}}\left(\cdot \right):R^{n}\times R^{n}\times R\to R_{+}$, i=1,2,…,N and mapping $\rho_{E_{i}}\left(\cdot \right):R^{n}\times R^{n}\times R\to R$, such that for all $x\in R^{n}$, $\sigma_{i}\in \Sigma_{i}$, i=1,2,…,N, $u\in R^{m}$ and t∈R,*

(38) $$\begin{array}{cc} \max_{\sigma_{i}\in \Sigma_{i}}∥h_{i}\left(z,x,\sigma_{i},t\right)∥ & \leq \rho_{h_{i}}\left(z,x,t\right), \end{array}$$

(39) $$\begin{array}{cc} \max_{\sigma_{i}\in \Sigma_{i}}∥E_{i}\left(z,x,\sigma_{i},t\right)∥ & \leq \rho_{E_{i}}\left(z,x,t\right), \end{array}$$

*where $\rho_{E_{i}}\left(z,x,t\right)<1$ for all t∈R.*

**Assumption** **3.** *For each i=1,2,…,N, $\Sigma_{i}$ is compact, and $\sigma_{i}$ is Lebesgue measurable.*

From those, the robust controller is designed:

(40) $$\begin{array}{cc} u_{i}= & \left\{\begin{array}{cc} u_{1i}+u_{2i},i<N & \\ u_{1i},i=N \end{array}\right. \end{array}$$

(41) $$\begin{array}{cc} u_{1i}= & \left\{\begin{array}{c} -\frac{B_{i}^{T}\left(x_{i},t\right)\nabla_{z_{i}}V_{i}\left(z_{i},t\right)\rho_{i}}{\left|B_{i}^{T}\left(x_{i}\left(t\right),t\right)\nabla_{z_{i}}V_{i}\left(z_{i},t\right)\rho_{i}\right|}\rho_{i},∥\mu_{i}∥>\epsilon_{i}\\ -\sin\left(\frac{\pi \mu_{i}}{2\epsilon_{i}}\right),∥\mu_{i}∥\leq \epsilon_{i} \end{array}\right. \end{array}$$

(42) $$\begin{array}{cc} \mu_{i}= & B_{i}^{T}\left(x_{i},t\right)\nabla_{z_{i}}V_{i}\left(z_{i},t\right)\rho_{i} \end{array}$$

(43) $$\begin{array}{cc} u_{2i}= & -\beta_{i}B_{i}^{T}\left(x_{i},t\right)\nabla_{x_{i}}V\left(x_{i},t\right), \end{array}$$

(44) $$\begin{array}{cc} \rho_{i}= & \frac{\rho_{h_{i}}\left(z,x,t\right)}{1-\rho_{E_{i}}\left(z,x,t\right)}, \end{array}$$

(45) $$\begin{array}{cc} u= & u_{N}. \end{array}$$

## 4. Stability Analysis

The stability analysis will be divided into two distinct phases. Firstly, we will prove the practical stability of state variables $z_{i},i=1,2,\ldots ,N$ of the system (33), under the control (40). Secondly, because of $z_{1}=x_{1}$, the state variable $x_{1}$ in system (31) will also exhibit practical stability.

**Theorem** **1.** *Consider dynamic system (33) which satisfy Assumptions 1, 2 and 3. Control $u_{i},i=1,2,\ldots ,N$ in (40) render the state of the system, x(t), practically stable:*

*(i)*  *Existence and Continuation of the solution: The system $\hat{S}$ possesses a solution $z\left(t\right):\left[t_{0},\infty \right)\to R^{n}$.*
*(ii)*  *Uniform boundedness: For any $r_{z}>0$, there exist a constant $d_{z}\left(r_{z}\right)>0$ such that if $∥z(t_{0})∥\leq r_{z}$, then $∥z(t)∥\leq d_{z}\left(r_{z}\right)$ for all $t\geq t_{0}$.*
*(iii)*  *Uniform ultimate boundedness: There exists d>0, such that for any $\bar{d}>d$, any $r_{z}>0$ and any z(t) with $∥z(t_{0})∥\leq r_{z}$, there exist a finite time $T(\bar{d},r_{z})$ such that $∥z(t)∥\leq \bar{d}$ for any $t\geq t_{0}+T\left(\bar{d},r_{z}\right)$.*

**Proof.**  Choose the Lyapunov function for the system as follows:

(46) $$\begin{array}{c} V=\sum_{i=1}^{N}V_{i}, \end{array}$$

where $V_{i}$ is the Lyapunov function mentioned in Assumption 1.

The gradient of the Lyapunov function with respect to the system yields:

(47) $$\begin{array}{c} L=\sum_{i=1}^{N}L_{i}=\sum_{i=1}^{N}\frac{dV_{i}}{dt}. \end{array}$$

Defining $\Phi_{i}:=\nabla_{z_{i}}^{T}V_{i}B_{i}$, thus from (47), when i=1,2,…,N−1,

(48) $$\begin{array}{cc} L_{i}:= & \frac{\partial V_{i}}{\partial t}+\nabla_{z_{i}}^{T}V_{i}f_{i}+\nabla_{z_{i}}^{T}V_{i}\left(\Delta f_{i}+B_{i}u_{i}+\Delta u_{i}\right)+\nabla_{z_{i}}^{T}V_{i}B_{i}z_{i+1}\\ \leq & -\gamma_{3i}{∥z_{i}∥}^{2}+∥\Phi_{i}∥h_{i}+\Phi_{i}u_{i1}+\Phi_{i}E_{i}u_{i1}-\left(1-\rho_{E_{i}}\right)\beta_{i}{∥\Phi_{i}∥}^{2}+\left(1+\rho_{E_{i}}\right)\Phi_{i}z_{i+1}. \end{array}$$

If $∥\mu_{i}∥>\epsilon_{i}$, there is

(49) $$\begin{array}{cc} L_{i}\leq & -\gamma_{3i}{∥z_{i}∥}^{2}+\Phi_{i}h_{i}-∥\Phi_{i}∥\frac{\rho_{h_{i}}}{1-\rho_{E_{i}}}+∥\Phi_{i}∥\frac{\rho_{h_{i}}}{1-\rho_{E_{i}}}\rho_{E_{i}}-\left(1-\rho_{E_{i}}\right)\beta_{i}{∥\Phi_{i}∥}^{2}+\left(1+\rho_{E_{i}}\right)\Phi_{i}z_{i+1}\\ \leq & -\gamma_{3i}{∥z_{i}∥}^{2}+\Phi_{i}h_{i}-∥\Phi_{i}∥\rho_{h_{i}}-\left(1-\rho_{E_{i}}\right)\beta_{i}{∥\Phi_{i}∥}^{2}+\left(1+\rho_{E_{i}}\right)\Phi_{i}z_{i+1}\\ \leq & -\gamma_{3i}{∥z_{i}∥}^{2}-\left(1-\rho_{E_{i}}\right)\beta_{i}{∥\Phi_{i}∥}^{2}+\left(1+\rho_{E_{i}}\right)\Phi_{i}z_{i+1}. \end{array}$$

Otherwise,

(50) $$\begin{array}{cc} L_{i}\leq & -\gamma_{3i}{∥z_{i}∥}^{2}+\Phi_{i}h_{i}+\Phi_{i}\sin\left(\frac{\pi \mu_{i}}{2\varepsilon }\right)\frac{\rho_{h_{i}}}{1-\rho_{E_{i}}}-\Phi_{i}\sin\left(\frac{\pi \mu_{i}}{2\varepsilon }\right)\frac{\rho_{h_{i}}}{1-\rho_{E_{i}}}E_{i}\\ \; & -\left(1-\rho_{E_{i}}\right)\beta_{i}{∥\Phi_{i}∥}^{2}+\left(1+\rho_{E_{i}}\right)\Phi_{i}z_{i+1}\\ \leq & -\gamma_{3i}{∥z_{i}∥}^{2}+\Phi_{i}h_{i}+\Phi_{i}\frac{\rho_{h_{i}}}{1-\rho_{E_{i}}}-\Phi_{i}\frac{\rho_{h_{i}}}{1-\rho_{E_{i}}}\rho_{E_{i}}-\left(1-\rho_{E_{i}}\right)\beta_{i}{∥\Phi_{i}∥}^{2}+\left(1+\rho_{E_{i}}\right)\Phi_{i}z_{i+1}\\ \leq & -\gamma_{3i}{∥z_{i}∥}^{2}+2\Phi_{i}\rho_{h_{i}}-\left(1-\rho_{E_{i}}\right)\beta_{i}{∥\Phi_{i}∥}^{2}+\left(1+\rho_{E_{i}}\right)\Phi_{i}z_{i+1}\\ \leq & -\gamma_{3i}{∥z_{i}∥}^{2}+2\epsilon_{i}-\left(1-\rho_{E_{i}}\right)\beta_{i}{∥\Phi_{i}∥}^{2}+\left(1+\rho_{E_{i}}\right)\Phi_{i}z_{i+1}. \end{array}$$

Therefore, from (49) and (50),

(51) $$\begin{array}{cc} L_{i}\leq & -\gamma_{3i}{∥z_{i}∥}^{2}+2\epsilon_{i}-\left(1-\rho_{E_{i}}\right)\beta_{i}{∥\Phi_{i}∥}^{2}+\left(1+\rho_{E_{i}}\right)\Phi_{i}z_{i+1}. \end{array}$$

Similarly, when i=N, there is

(52) $$\begin{array}{cc} L_{N}\leq & -\gamma_{3N}{∥z_{N}∥}^{2}+2\epsilon_{i}. \end{array}$$

Therefore, from (47), (51) and (52),

(53) $$\begin{array}{cc} L\leq & -\sum_{i=1}^{N}\gamma_{3i}{∥z_{i}∥}^{2}+2\sum_{i=1}^{N}\epsilon_{i}-\sum_{i=1}^{N-1}\left(1-\rho_{E_{i}}\right)\beta_{i}{∥\Phi_{i}∥}^{2}+\sum_{i=1}^{N-1}\left(1+\rho_{E_{i}}\right)\Phi_{i}z_{i+1}\\ \leq & -\sum_{i=1}^{N}\gamma_{3i}{∥z_{i}∥}^{2}+2\sum_{i=1}^{N}\epsilon_{i}-\sum_{i=1}^{N-1}\beta_{i}\left(1-\rho_{E_{i}}\right)\\ \; & \cdot \left[{∥\Phi_{i}∥}^{2}-2\frac{\left(1+\rho_{E_{i}}\right)}{2\beta_{i}\left(1-\rho_{E_{i}}\right)}\Phi_{i}z_{i+1}+\frac{{\left(1+\rho_{E_{i}}\right)}^{2}}{4\beta_{i}^{2}{\left(1-\rho_{E_{i}}\right)}^{2}}{∥z_{i+1}∥}^{2}-\frac{{\left(1+\rho_{E_{i}}\right)}^{2}}{4\beta_{i}^{2}{\left(1-\rho_{E_{i}}\right)}^{2}}{∥z_{i+1}∥}^{2}\right]\\ \leq & -\sum_{i=1}^{N}\gamma_{3i}{∥z_{i}∥}^{2}+2\sum_{i=1}^{N}\epsilon_{i}-\sum_{i=1}^{N-1}\beta_{i}\left(1-\rho_{E_{i}}\right){\left[∥\Phi_{i}∥-\frac{\left(1+\rho_{E_{i}}\right)}{2\beta_{i}\left(1-\rho_{E_{i}}\right)}z_{i+1}\right]}^{2}\\ \; & +\sum_{i=1}^{N-1}\beta_{i}\left(1-\rho_{E_{i}}\right)\frac{{\left(1+\rho_{E_{i}}\right)}^{2}}{4\beta_{i}^{2}{\left(1-\rho_{E_{i}}\right)}^{2}}{∥z_{i+1}∥}^{2}\\ \leq & -\sum_{i=1}^{N}\gamma_{3i}{∥z_{i}∥}^{2}+2\sum_{i=1}^{N}\epsilon_{i}+\sum_{i=1}^{N-1}\frac{{\left(1+\rho_{E_{i}}\right)}^{2}}{4\beta_{i}\left(1-\rho_{E_{i}}\right)}{∥z_{i}∥}^{2}\\ \leq & -\sum_{i=1}^{N-1}\left[\gamma_{3i}-\frac{{\left(1+\rho_{E_{i}}\right)}^{2}}{4\beta_{i}\left(1-\rho_{E_{i}}\right)}\right]{∥z_{i}∥}^{2}-\gamma_{3N}{∥z_{N}∥}^{2}+2\sum_{i=1}^{N}\epsilon_{i}. \end{array}$$

Define

(54) $$\begin{array}{cc} {\hat{\gamma }}_{1}\left(v\right):= & \min_{z}\left\{\gamma_{1i}\left(∥z_{i}∥\right)|v=∥z∥,i=1,2,\ldots ,N\right\}, \end{array}$$

(55) $$\begin{array}{cc} {\hat{\gamma }}_{2}\left(v\right):= & \max_{z}\left\{\gamma_{2i}\left(∥z_{i}∥\right)|v=∥z∥,i=1,2,\ldots ,N\right\}, \end{array}$$

(56) $$\begin{array}{cc} {\hat{\gamma }}_{3}:= & \min\left\{\gamma_{3i}-\frac{{\left(1+\rho_{E_{i}}\right)}^{2}}{4\beta_{i}\left(1-\rho_{E_{i}}\right)},\gamma_{3_{N}}|i=1,2,\ldots ,N-1\right\}, \end{array}$$

(57) $$\begin{array}{cc} \delta := & 2\sum_{i=1}^{N}\epsilon_{i}, \end{array}$$

thus from (53) and Assumption 1, results of [15,27] guarantee that:

(i) Existence and Continuation of the solution: From [28], the system *S* possesses a solution $x_{1}\left(t\right):\left[t_{0},\infty \right)\to R^{n_{1}}$.
(ii) Uniform boundedness: For any $r_{z}>0$, if $∥x_{1}\left(t_{0}\right)∥\leq r_{z}$, then there exists:
  (58) $$\begin{array}{c} d_{z}\left(r_{z}\right)=\left\{\begin{array}{c} \left({\hat{\gamma }}_{1}^{-1}\circ {\hat{\gamma }}_{2}\right)\left(r_{z}\right),\mathrm{if}\;r_{z}>R_{z},\\ \left({\hat{\gamma }}_{1}^{-1}\circ {\hat{\gamma }}_{2}\right)\left(R_{z}\right),\mathrm{otherwise} \end{array}\right. \end{array}$$
  where
  (59) $$\begin{array}{c} R_{z}=\sqrt{\delta {\hat{\gamma }}_{3}^{-1}}, \end{array}$$
  such that for $t\geq t_{0}$, $∥\varsigma (t)∥\leq d_{z}\left(r_{z}\right)$.
(iii) Uniform ultimate boundedness: There exists $d=\left({\hat{\gamma }}_{1}^{-1}\circ {\hat{\gamma }}_{2}\right)\left(R_{z}\right)$, such that for any $\bar{d}>d$, any $r_{z}>0$ and any $x_{1}\left(t\right)$ with $∥x_{1}\left(t_{0}\right)∥\leq \overset{¯}{\bar{d}}$, there exist
  (60) $$\begin{array}{c} \begin{array}{c} T_{z}\left(\bar{d},r_{z}\right)=\left\{\begin{array}{cc} 0, & \mathrm{if}\;\;r_{z}\leq \bar{R}\\ \frac{{\hat{\gamma }}_{2}\left(r_{z}\right)-\left({\hat{\gamma }}_{1}\circ {\hat{\gamma }}_{2}^{-1}\circ {\hat{\gamma }}_{1}\right)\left(\bar{d}\right)}{{\hat{\gamma }}_{3}{\left[\left({\hat{\gamma }}_{2}^{-1}\circ {\hat{\gamma }}_{1}\right)\left(\bar{d}\right)\right]}^{2}-\delta }, & \mathrm{otherwise} \end{array}\right., \end{array} \end{array}$$
  such that $∥x_{1}\left(t\right)∥\leq \bar{d}$ for any $t\geq t_{0}+T\left(\bar{d},r\right)$, where
  (61) $$\begin{array}{c} \bar{R}=\left({\hat{\gamma }}_{2}^{-1}\circ {\hat{\gamma }}_{1}\right)\left(\bar{d}\right). \end{array}$$

Therefore, Theorem 1 has been proven. □

**Remark** **2.** *Notably, the radius of the uniform ultimate ball d is directly proportional to δ, ${\hat{\gamma }}_{1}$, ${\hat{\gamma }}_{2}^{-1}$ and ${\hat{\gamma }}_{3}^{-1}$. This means smaller steady-state error can be achieved by adjusting all parameters related to δ, ${\hat{\gamma }}_{1}$, ${\hat{\gamma }}_{2}^{-1}$ and ${\hat{\gamma }}_{3}^{-1}$ to make them smaller. Similarly, adjusting parameters related to Tz to make it smaller can shorten the set time.*

Theorem 1 guarantees the practical stability performance of the transformed system (33). Since $z_{1}=x_{1}$, if the state variable $z_{1}$ in the system (33) described by (32) has practical stability under the control *u* in (40), then the state variable $x_{1}$ in the system (6) will also exhibit practical stability under the same control *u* in (40).

**Remark** **3.** *The control design procedure can be summarized as follows:*

*Step* *1:*  *Let i=1, Model the dynamics of system S as (31).*
*Step* *2:*  *Define the tracking errors $z_{i}$ as (32). Then rewrite the dynamic equations in the state-space form as (33).*
*Step* *3:*  *Design $f_{i}\left(\cdot \right)$ and $V_{i}\left(\cdot \right)$ in (33) and (40) according to Definition 1.*
*Step* *4:*  *Discuss the bounding condition of the uncertain portion $\Delta f_{i}\left(\cdot \right)$ and $\Delta B_{i}\left(\cdot \right)$ in (33) to yield $\rho_{h_{i}}\left(\cdot \right)$ as (38) and $\rho_{E_{i}}\left(\cdot \right)$ as (39).*
*Step* *5:*  *Chose $\beta_{i}$ and $\epsilon_{i}$. Then the implanted control $u_{i}$ is given by (40).*
*Step* *6:*  *If i=N, stop. Otherwise, Let i=i+1 and back to Step 2.*

**Figure 3:**
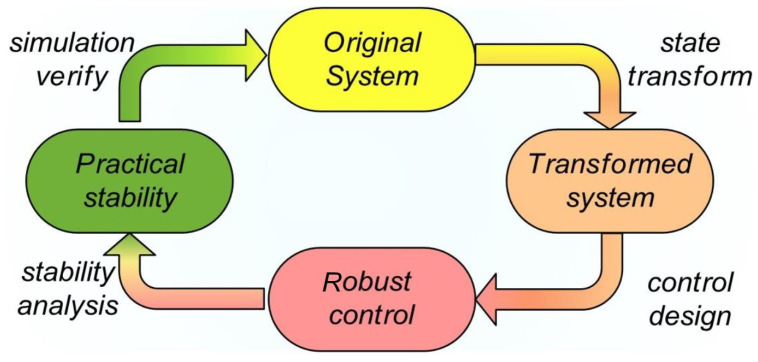
Loop of control design and stability proof.

Through the above process, we can leverage the system’s dynamic model to design the controller step by step starting from the virtual control $u_{1}$, ultimately obtaining the controller $u=u_{N}$. We depict the transformation-based control design and performance analysis loop as Figure 3.

## 5. Feasibility Verification

Section 5 will verify that the system (7) satisfies all assumptions and is a particular case of (31), thus the conclusions in Theorem 1 can also apply to (7).

Firstly, as a particular case of the latter of the system scripted by (31), the system scripted by (7) has the same formulation.

Secondly, if we choose $k_{11},k_{12}$, $k_{2}$ and $k_{3}\in R_{+}$, Assumption 1 will be satisfied.

Thirdly, from (10), (19) and (25), $\rho_{h_{1}}$, $\rho_{E_{1}}$, $\rho_{h_{2}}$, $\rho_{E_{2}}$, $\rho_{h_{3}}$ and $\rho_{E_{3}}$ could be chosen:

(62) $$\begin{array}{cc} \rho_{h_{1}}= & \left|k_{11}x_{11}+k_{12}x_{12}-m{\ddot{y}}_{d}-B_{p}\dot{y}-{\hat{F}}_{f}\right|+{\hat{F}}_{f}+\hat{F}, \end{array}$$

(63) $$\begin{array}{cc} \rho_{E_{1}}= & 0, \end{array}$$

(64) $$\begin{array}{cc} \rho_{h_{2}}= & \frac{\left|k_{2}x_{2}-\left(L_{1}S_{1}+L_{2}S_{2}\right)\dot{y}\right|}{L_{1}+L_{2}}+\frac{\left|L_{1}\right|{\hat{Q}}_{c1}-\left|L_{2}\right|{\hat{Q}}_{c2}+\hat{Q}}{L_{1}+L_{2}}+{\hat{d}}_{q}, \end{array}$$

(65) $$\begin{array}{cc} \rho_{E_{2}}= & 0, \end{array}$$

(66) $$\begin{array}{cc} \rho_{h_{3}}= & \frac{J_{a}}{{\bar{k}}_{m}}\left|k_{3}x_{3}-\frac{x_{3}}{J_{a}}\right|+\frac{{\hat{T}}_{b}}{{\hat{k}}_{m}}, \end{array}$$

(67) $$\begin{array}{cc} \rho_{E_{3}}= & \frac{{\hat{k}}_{m}}{{\bar{k}}_{m}}, \end{array}$$

where ${\hat{F}}_{f}>\max_{t}\left\{\Delta F_{f}\left(y\left(t\right),\dot{y}\left(t\right),t\right)|t\in R\right\},{\hat{F}}_{t}>\max_{t}\left\{\Delta F_{t}\left(t\right)|t\in R\right\}$, ${\hat{Q}}_{c1}>\max_{t}\left\{\Delta Q_{c1}\left(t\right)|t\in R\right\}$, ${\hat{Q}}_{c2}>\max_{t}\left\{\Delta Q_{c2}\left(t\right)|t\in R\right\}$, ${\hat{d}}_{q}>\max_{t}\left\{\Delta d_{q}\left(t\right)|t\in R\right\}$, $\hat{Q}>\max_{t}\left\{\Delta Q(t)|t\in R\right\}$ and ${\hat{T}}_{b}>\max_{t}\left\{\Delta T_{b}\left(t\right)|t\in R\right\}$ are constants. Thus Assumption 2 and Assumption 3 are satisfied when ${\bar{k}}_{m}>0$.

In conclusion, system (7) will be a specific instance of (31) and satisfies Assumptions 1 and 2 when ${\bar{k}}_{m}>0$. As a result, results of Theorems 1 also apply to (7).

## 6. Simulink-Amesim Co-Simulation

Using numerical simulation, the superiority of the proposed Robust Control (RC) will be thoroughly confirmed in Section 6. Using the Simulink-Amesim co-simulation platform, the simulation was run. The electrical-hydrostatic actuator is modeled using Amesim, a hydraulic simulation platform, and the control algorithm is coded using Simulink, a numerical computing program. A fixed 0.01 s step size is used for the simulation.

For comparison, the fixed-gain Sliding Mode Controller (SMC) with linear sliding surface and the $H_{2}/H_{\infty }$ Hybrid Control (HTI) are employed. The linearized model without disturbance adopts

(68) $$\left[\begin{array}{c} {\dot{z}}_{11}\\ {\dot{z}}_{12} \end{array}\right]=\left[\begin{array}{cc} 0 & 1\\ -0.0742 & -11.6501 \end{array}\right]\left[\begin{array}{c} z_{11}\\ z_{12} \end{array}\right]+\left[\begin{array}{c} 0\\ 0.0198 \end{array}\right]u,$$

(69) $$\left[\begin{array}{c} y_{11}\\ y_{12} \end{array}\right]=\left[\begin{array}{c} z_{11}\\ z_{12} \end{array}\right],$$

HTI adopting the form of state feedback control $u=-k_{1h}z_{11}-k_{2h}z_{12}$, where $k_{1h},k_{2h}\in R$. Since high-frequency vibrations may render the physical model ineffective, SMC is altered with an anti-vibration treatment and can be expressed as

(70) $$\begin{array}{c} u_{1}=\left\{\begin{array}{c} -k_{1s}\mathrm{sgn}\left(k_{2s}z_{11}+k_{3s}z_{12}\right),\;\mathrm{if}\;\left|k_{2s}z_{11}+k_{3s}z_{12}\right|>\epsilon \\ -k_{1s}\left(k_{2s}z_{11}+k_{3s}z_{12}\right)/\epsilon ,\;\mathrm{otherwise} \end{array}\right. \end{array}$$

where $k_{1s},k_{2s},k_{3s}\in R$ and ϵ>0 and sgn(·) is the signal function. It was selected because its direction and extent of control are intuitively apparent and is simple to apply.

**Figure 4:**
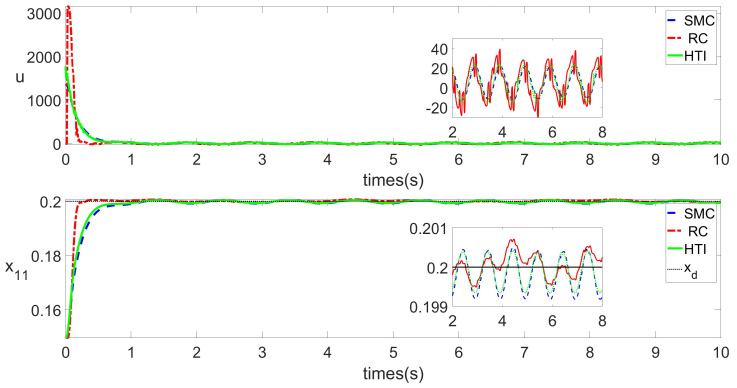
*u* and $x_{11}$ under RC and SMC in Scenario 1.

**Figure 5:**
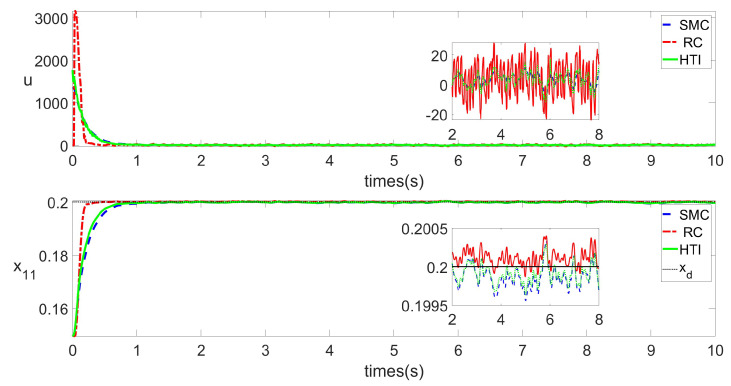
*u* and $x_{11}$ under RC and SMC in Scenario 2.

**Figure 6:**
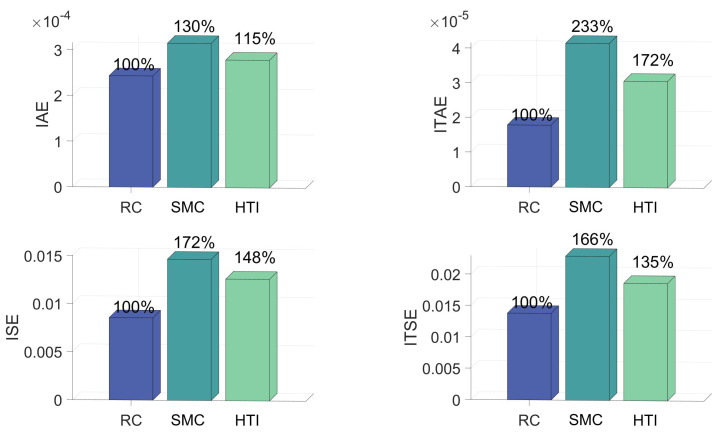
Performance metrics under RC and SMC in Scenario 1.

**Figure 7:**
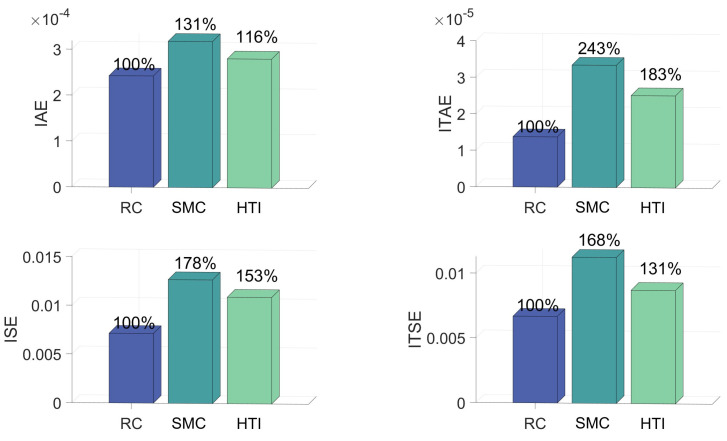
Performance metrics under RC and SMC in Scenario 2.

In this study, two control situations are taken into account. The impact of low-frequency uncertainty on the $C^{2}$ step reference signal is taken into account in Scenario 1. Scenario 2 takes into account how uncertainty at low and high frequencies affect the $C^{2}$ step reference signal. Figure 4, Figure 5, Figure 6 and Figure 7 illustrate the changes in a few key simulation parameters. Control and physical parameters are shown in Table 1 and Table 2, where σ∈[−1,1] is the uncertainty variable.

In Scenario 1, Figure 4 compares the variation of the rod displacement over time under the action of SMC, HTI and RC. By comparing the state variable $x_{11}$, we can see that from 0 s to around 0.8 s, RC respond more quickly than SMC and HTI. However, RC responds more intensely, which makes it stabilize slightly faster (around 0.2 s). After 0.8 s, systems controlled by both algorithms are basically stable, with steady-state errors at the same level. Comparing the control input *u*, it can be observed that the RC algorithm has a larger magnitude of change and also tends to stabilize more quickly before 0.8 s. This is consistent with the trend of $x_{11}$.

In Scenario 2, there are more complex disturbances, such as more complex aerodynamic forces. Results are presented in Figure 5. All the figures exhibit a sawtooth shape, indicating more severe disturbances within the system compared to Scenario 1. Nevertheless, similar to SMC and HTI, RC has not been significantly affected by excessive noise, even without the use of filters. Comparing the size of the state variable $x_{11}$ from Figure 5, it can be seen that RC responds quickly and stabilizes at 0.2 s, while SMC and HTI are stable at 1 s. Steady-state errors are at the same level. Comparing the control input *u*, it can be observed that although there are more complex uncertainties, the RC algorithm has a larger magnitude of change at the beginning and also tends to stabilize more quickly. This indicates that RC is not a fragile algorithm.

Figure 6 and Figure 7 quantify the control process and present four control metrics: integral of squared error (ISE), integral of time-weighted squared error (ITSE), integral of absolute error (IAE) and integral of time-weighted absolute error (ITAE). ISE represents the level of error oscillations throughout the control process. ITSE represents the system’s steady-state oscillations. IAE represents a balanced performance indicator of the entire control process. ITAE represents the system’s steady-state error. Figure 6 indicates that among all metrics in Scenario 1, RC exhibits better control performance than SMC and HTI, particularly in terms of ITAE. IAE of SMC is about 30% more than RC, while ITAE of SMC is 133% more than RC, ISE of SMC is 72% more than RC and ITSE of SMC is 66% more than RC. IAE of HTI is about 15% more than RC, while ITAE of HTI is 72% more than RC, ISE of HTI is 48% more than RC and ITSE of HTI is 35% more than RC.

Figure 7 indicates that among all metrics in Scenario 2, and conclusions in Scenario 1 still hold. We can see that IAE of SMC is about 31% more than RC, while ITAE of SMC is 143% more than RC, ISE of SMC is 78% more than RC and ITSE of SMC is 68% more than RC. IAE of HTI is about 16% more than RC, while ITAE of HTI is 83% more than RC, ISE of HTI is 53% more than RC and ITSE of HTI is 31% more than RC. Specifically, we found that the more complex uncertainty, namely Scenario 2, further intensified this phenomenon. This indicates that RC is better at handling complex uncertainties.

Through the above various metrics, we can see that regardless of the control scenario or the metric used, the performance of RC is superior to the two comparative algorithms. Furthermore, in systems with stronger uncertainties, the performance gap between RC and the comparative algorithms widens even more, indicating that RC has a greater performance advantage under conditions of higher uncertainty level. This means that RC is highly suitable for the control scenario of aero-engine variable stator vanes: with stronger uncertainties and higher performance requirements.

## 7. Conclusions

This paper proposes a Robust Control (RC) based on the backstepping approach to achieve precise control under complex uncertainties through multi-loop control, addressing the issue of accurate position control in the stator vane control system of aero-engines. The control performance of this method and the correlation between control parameters and performance are analyzed at the theoretical level. Finally, this control method was subjected to numerical hydraulic simulation in two working environments and compared with Sliding Mode Control (SMC) and $H_{2}/H_{\infty }$ Hybrid control (HTI). Four different performance metrics were used for comparison. The results show that under two working environments and across four performance metrics, RC demonstrated a performance advantage over SMC ranging from a minimum of 24% to a maximum of 123%, and a performance advantage over HTI ranging from a minimum of 15% to a maximum of 83%. This means that under any circumstances and performance metrics, the control error of RC is significantly lower than that of SMC, showing better control effectiveness.

## Figures and Tables

**Table 1:** Physical parameters.

Name	Value	Unit
$S_{1}$	0.00589	m
$S_{2}$	0.00589	m
*m*	2	kg
*m*	2	kg
$F_{f}$	1400	N
$B_{e}$	1650	MPa
$V_{0}$	0.000050	m^3^
$k_{q}$	5.2	-
*l*	0.3	m
$J_{\alpha }$	1	kg/m^2^
$k_{m}$	1.9	N·s/m

**Table 2:** Control parameters.

Name	Value	Name	Value
$k_{s1}$	8000	$k_{11}$	140,000
$k_{s2}$	1	$k_{12}$	100
$k_{s3}$	0.1	$k_{2}$	10
ϵ	0.1	$k_{3}$	0.005
$k_{h1}$	35,440	$\epsilon_{1}$	0.35
$k_{h2}$	851	$\epsilon_{2}$	0.01
$\rho_{1}$	1000	$\epsilon_{3}$	5
$\rho_{2}$	300		

## Data Availability

The data and code of the current study can be obtained from the corresponding author upon reasonable request.
